# Quantitative proteomic profiling of Cervicovaginal fluid from pregnant women with term and preterm birth

**DOI:** 10.1186/s12953-021-00171-1

**Published:** 2021-02-15

**Authors:** Young Eun Kim, Kwonseong Kim, Han Bin Oh, Sung Ki Lee, Dukjin Kang

**Affiliations:** 1 Center for Bioanalysis, Division of Chemical and Medical Metrology, Korea Research Institute of Standards and Science, 267 Gajeong-Ro, Yuseong-Gu, Daejeon, 34113 South Korea; 2 Department of Chemistry, Sogang University, Seoul, 04107 South Korea; 3 Department of Obstetrics and Gynecology, Konyang University Hospital, 158 Gasuwondong-Ro, Seo-Gu, Daejeon, 3535 South Korea

**Keywords:** Cervicovaginal fluid, Preterm birth, Quantitative proteomics

## Abstract

**Background:**

Preterm birth (PTB) is one of major causes of perinatal mortality and neonatal morbidity, but knowledge of its complex etiology is still limited. Here we present cervicovaginal fluid (CVF) protein profiles of pregnant women who subsequently delivered at spontaneous preterm or term, aiming to identify differentially expressed CVF proteins in PTB and term birth.

**Methods:**

The CVF proteome of women who sequentially delivered at preterm and term was analyzed using isobaric tags for relative and absolute quantitation (iTRAQ) coupled with two-dimensional nanoflow liquid chromatography-tandem mass spectrometry (2D-nLC-MS/MS). We compared the CVF proteome of PTB (*n* = 5) and control subjects (term birth, *n* = 7) using pooled control CVF (term birth, *n* = 20) as spike-in standard.

**Results:**

We identified 1294 CVF proteins, of which 605 were newly identified proteins. Of 990 proteins quantified in both PTB and term birth, 52 proteins were significantly up/down-regulated in PTB compared to term birth. The differentially expressed proteins were functionally associated to immune response, endopeptidase inhibitors and structural constituent of cytoskeleton. Finally, we confirm the down-regulation of SERPINB7 (a serine-type protease inhibitor) in PTB compared to control by Western blot.

**Conclusions:**

Taken together, our study provide quantitative CVF proteome profiles of pregnant women who ultimately delivered at preterm and term. These promising results could help to improve the understanding of PTB etiology and to discover biomarkers for asymptomatic PTB.

**Supplementary Information:**

The online version contains supplementary material available at 10.1186/s12953-021-00171-1.

## Background

Preterm birth (PTB) is defined as delivery before 37 weeks of pregnancy. Early diagnosis and interventions of PTB are important issues to reduce neonatal morbidity and mortality [1], but the clinical utility of predictive markers for PTB is still limited. The ultrasound assessment of cervical length is a widely accepted method to determine the risk for PTB. Although short cervical length is correlated with the frequency of PTB, it alone is not sufficient to achieve the predictive accuracy of PTB [2–4]. Therefore, a combination of biophysical and biochemical tests has been tried in an attempt to improve their predictive capacity [4–7].

The fetal fibronectin (fFN) test is the most clinically useful assay for the assessment of risk for PTB, which measures the level of fFN in cervicovaginal fluid (CVF). The fFN, a glycoprotein in the extracellular matrix, is commonly not detected in CVF after approximately 20 weeks of gestation due to their tight interactions at the maternal-fetal interface [8]. The presence of fFN in CVF after 20 weeks of gestation indicates the disruption of the maternal-fetal attachments, so the high level of fFN (over the 50 ng/mL) is considered to be at a high risk of PTB. Although fFN test has good predictive value in symptomatic women, it has poor sensitivity in asymptomatic women [4, 7]. There is a significant need for the development of alternative biomarkers that predict PTB even in asymptomatic population and early stages of gestation (before 20 weeks) in asymptomatic women.

Mass spectrometry (MS)-based proteomics has become a promising technology for the discovery of new biomarkers [9–13]. Recent advances on multidimensional liquid chromatography (LC)-tandem mass spectrometry (MS/MS) have allowed the large-scale profiling of proteins from complex biological samples [14, 15]. Diverse isotope-labeling strategies (e.g. SILAC, mTRAQ, iTRAQ and TMT) are currently used for the quantitative proteomics, which provide valuable information on the proteome alterations between several different biological samples [16, 17]. In the initial step of clinical approach, furthermore, pooling individual proteome samples as a spike-in standard allows to reduce biological variation, thereby increasing the reliability in quantitative proteomic datasets [18–20]. For example, the super-SILAC strategy use combining mixtures of different SILAC-labeled cell line as a spike-in standard, enabling more accuracy and multiplexed profiling for various types of tissues and clinical samples [19–21].

Various types of biological fluids have been subjected to discover biomarkers for the prediction of PTB, such as serum, amniotic fluid, CVF and urine [5–7]. Serum/plasma is mostly used to excavate biomarker for clinical diagnosis [13, 22–24]. However, there are some hurdles such as dilution and low organ specificity of biomarkers resulted from large volume/organ ratio and blood circulation throughout entire body. On the contrary, CVF is a dynamic fluid composed of proteins and other substances from vagina, cervix, and uterine that provide more specific information for female reproductive system compared to other body fluids [25]. Also, due to its relatively small volume/organ ratio, the possibility of dilution of biomarkers is lower compared to serum/plasma samples. Therefore, CVF is a promising source of diagnostic information on both maternal and fetal health during pregnancy [4, 25, 26]. So far, there are relatively few proteomic studies that have focused on the quantitative profiling of PTB-driven CVF proteome [27–29]. Pereira et al. have previously reported the label-free quantitative analysis of CVF proteome from PTB and preterm labour (PTL) group [27]. They identified a total of 205 CVF proteins and a number of PTB/PTL-related proteins, including fibronectin, S100 proteins, acute-phase reaction proteins and cytoskeletal proteins. In another study, Lo et al. also have shown the comparative profiling of CVF proteins in asymptomatic women with a history of PTB based on the label-free quantification [28]. Of 748 proteins identified, four candidate proteins involved in immune and inflammatory response were proposed as biomarkers of PTB.

Herein, the comparative quantitative profiling of the CVF proteome were performed to identify differentially expressed proteins in PTB and term birth. CVF specimen was collected before 20 weeks of gestation. While most previous proteomic studies for the assessment of clinical samples are performed on pooled specimens, we compared proteome of CVF from individual PTB (*n* = 5) and individual control subjects (term birth, *n* = 7) using a spike-in standard (pooled CVF sample from 20 individual term birth subjects). The isobaric tags for relative and absolute quantitation (iTRAQ) combined with two-dimensional nanoflow liquid chromatography-tandem mass spectrometry (2D-nLC-MS/MS) approach was applied, resulting in the large-scale identification of CVF proteins including 605 newly identified proteins. Quantitative profiling of CVF proteins revealed the significant difference of CVF proteome between PTB and term birth group. In particular, proteins involved in immune response, endopeptidase inhibitors and structural constituent of cytoskeleton were differentially expressed in PTB compared to term birth group.

## Methods

### Subject recruitment

**Fig. 1:**
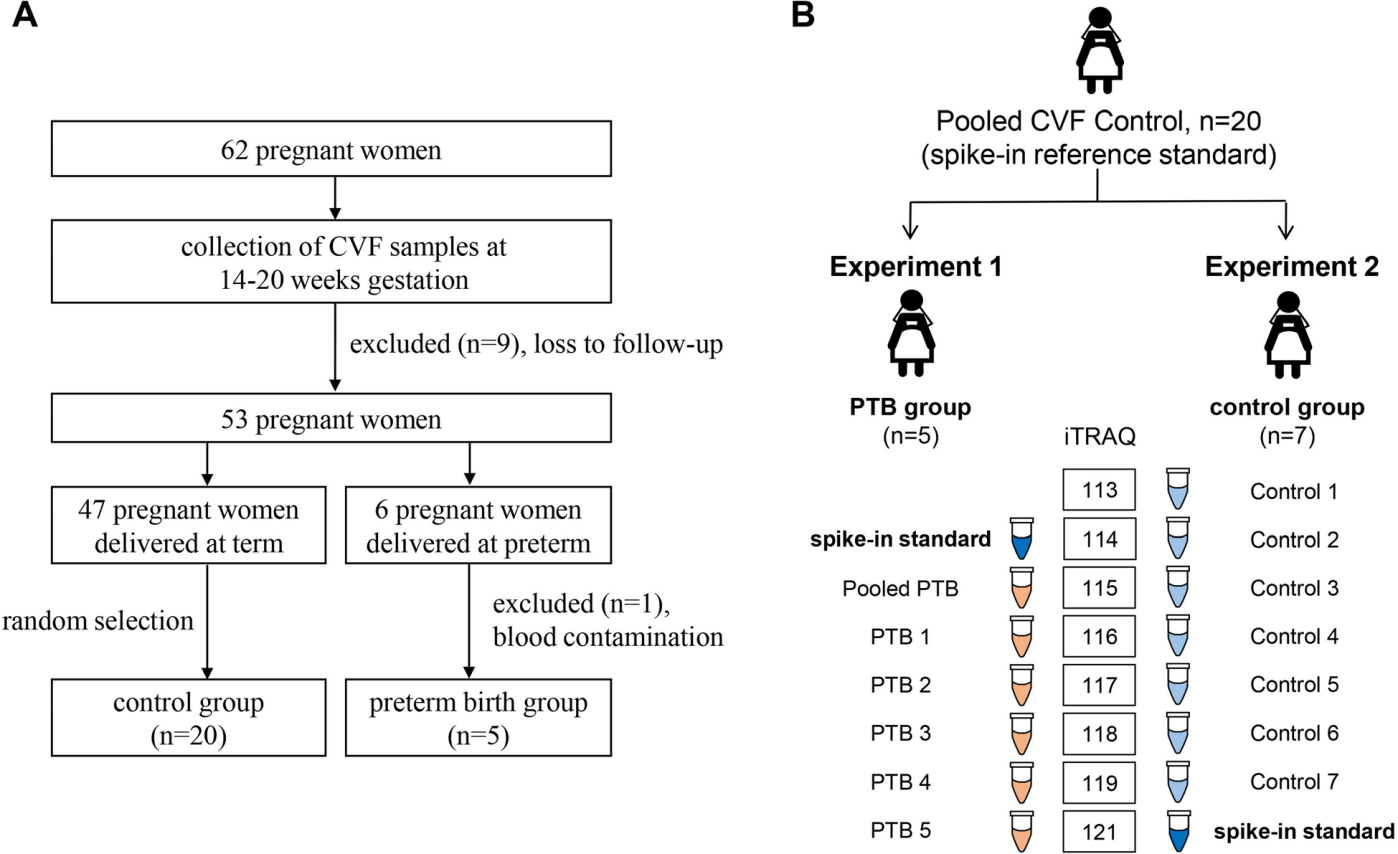
Study design and experimental workflow. Study design **a** and workflow **b** for quantitative proteomic analysis of CVF from PTB and term birth (control) group

This prospective observational study was approved by local research ethics committee and all participants signed informed consent before enrollment. All participants were recruited at Konyang University Hospital (Korea). Exclusion criteria for participant recruitment in the study were as follow: threatened abortion; hypertension; diabetes mellitus; multifetal pregnancy; Müllerian anomaly; and incompetence of cervix. A total of 62 pregnant women participated in the study, which of 9 were lost to follow-up (Fig. 1a). The remaining 53 participants were followed up until delivery: 47 who delivered at term (control group) and 6 who delivered spontaneously preterm (PTB group). In PTB group, one participant was excluded from further analysis due to the contamination of blood during CVF collection process. Statistical analysis was performed using R Statistics (version 2.11.1, The R Foundation for Statistical Computing, Vienna, Austria). Mann-Whitney U test was used to compare clinical parameters between term birth and PTB group.

### Sample collection

The CVF samples were collected between 14 and 20 weeks of gestation. The vagina was gently exposed with a vaginal speculum and CVF was obtained from the posterior fornix of the vagina by repeated irrigation and suction with a plastic pipette filled with 3 mL of phosphate buffered saline (PBS). We tried not to touch the cervix to avoid bleeding from the cervix. The specimens were stored in − 80 °C deep freezer or liquid nitrogen until analysis.

### Tryptic digestion and iTRAQ labeling procedure

The pooled control CVF sample as a reference standard was prepared from 20 term birth individual subjects by taking of equal amount of CVF protein in each sample. Equal amount of proteins from 5 PTB individual CVF samples were also pooled into one tube. A 25 μg of protein in each samples was used for proteomic analysis. Proteins were denatured with 6 M urea and 10 mM dithiothreitol (DTT) and alkylated with 20 mM iodoacetamide (IAA) for 30 min at room temperature in dark. The remaining IAA was reacted with the excess L-cysteine for 30 min at room temperature. The mixtures were then diluted to a final concentration of 1 M urea with 50 mM ammonium bicarbonate, digested with trypsin (1:50, w/w) for 18 h at 37 °C. The resulting tryptic peptides were desalted on Oasis HLB cartridge (Waters, Milford, U.S.A.) and dried using a Speed-Vac concentrator.

The iTRAQ labeling was performed according to the manufacturer’s instruction. We performed two independent iTRAQ experiments to compare the CVF proteome of individual PTB and individual control (term birth) subjects (Fig. 1b). The pooled CVF sample obtained from 20 individual term birth subjects was used as a spike-in reference standard for both Experiment 1 and 2. In Experiment 1, 5 individual PTB CVF samples and pooled control CVF sample were labeled with iTRAQ tags (114 tag for pooled controls, 115 tag for pooled PTB and 116, 117, 118, 119 and 121 tag for PTB individuals, respectively). After reaction for 2 h at room temperature, the resulting peptides were combined, desalted on Oasis HLB cartridge and dried. To consider individual variations in control group, randomly selected 7 individual CVF samples of control group were compared to pooled control CVF sample in Experiment 2 (113,114,115,116, 117, 118 and 119 tag for individual controls, respectively and 121 tag for pooled control). Each iTRAQ set of Experiment 1 and 2 was analyzed in a triplicate online 2D-nLC-MS/MS runs.

### Online 2D-nLC-MS/MS

Online 2D-nLC-MS/MS was performed using a 1260 capillary LC system (Agilent Technologies, Waldbronn, Germany) interfaced with Q Exactive™ Hybrid-Quadrupol-Orbitrap mass spectrometer (Thermo Fisher Scientific). For online 2D-nLC, biphasic trapping columns (4 cm in length) were packed in 200 μm-i.d. capillary with 5 mm of C18 resins (5 μm-200 Å, Apex Scientific, Maynooth, Ireland) followed by 15 mm of strong cation exchange (SCX) resins (5 μm-200 Å, Waters, Milford, U.S.A.). The reverse-phase (RP) analytical column (150 mm in length, 75 μm-i.D.) was packed with C18 resins (3 μm-100 Å, Bonna-Agela Technologies, Wilmington, U.S.A). The iTRAQ-labeled peptides were loaded on biphasic trapping column and eluted with following 12-step salt gradients: (1) 0 mM (2) 20 mM (3) 24 mM (4) 26 mM (5) 28 mM (6) 30 mM (7) 35 mM (8) 40 mM (9) 60 mM (10) 100 mM (11) 200 mM (12) 1000 mM ammonium bicarbonate in 0.1% formic acid. The eluted peptides were directly bound on the RP resin and then followed by binary gradient elution for RP-LC with buffer A (0.1% FA in water) and buffer B (2% water and 0.1% FA in acetonitrile). The RP-LC chromatography was carried out with a column flow rate of 200 nL/min. The mobile phase was held at 2% buffer B for 10 min, followed by 2 to 8% buffer B for 0.5 min, 8 to 15% buffer B for 4.5 min, 15 to 30% buffer B for 70 min, 30 to 90% buffer B for 3 min, 90% buffer B for 15 min, 90 to 2% buffer B for 2 min and 2% buffer B for 15 min.

The Q Exactive™ Hybrid-Quadrupole-Orbitrap mass spectrometer was operated in data-dependent mode. Full MS scans were acquired with an *m/z* ranges from 300 to 1800 at a resolution of 70,000. The automatic gain control (AGC) target values was set to 3 × 10^6^ with maximum injection times of 80 ms. For MS/MS scan, the 12 most intense precursor ions were selected and fragmented by high-energy collision dissociation (HCD) with a normalized collision energy (NCE) of 27%. The resolution of MS/MS scan was 35,000. Dynamic exclusion duration was set to 30 s. All iTRAQ-labeled samples were online 2D-nLC runs in technical triplicates.

### Data analysis and bioinformatics

The acquired raw files were searched using MaxQuant search engine 1.6.1.0 against the uniprot human database (Jan 3, 2018 release; 71,585 entries) for protein identification and iTRAQ quantification [30]. Two missed trypsin cleavage sites were allowed. The precursor mass tolerance value was set to 20 ppm for first search and 4.5 ppm for main search. Carbamidomethylation of cysteine (+ 57.021 Da) was set as fixed modifications and variable modifications were selected as follows: iTRAQ modification of N-terminal residue (+ 304.205 Da), iTRAQ modification of lysine (+ 304.205 Da), acetylation of N-terminal residue (+ 42.011 Da) and oxidation of methionine (+ 15.995 Da). Protein identification was accepted at false discovery rate (FDR) of protein and peptide less than 1%.

Data processing and statistical analysis were performed using the Perseus software 1.5.8.5 [31]. Identifications from the reverse decoy database and identified by site only were excluded. The iTRAQ ratios were log_2_ transformed and normalized by subtracting the median. The differentially expressed proteins were determined with a fold-change cut off of 1.5. For statistical analysis, two sample student’s t-test analysis was performed and the Benjamini-Hochberg procedure was subsequently applied to control for multiple testing [32]. The method of Benjamini-Hochberg was performed using R Statistics (version 4.0.2, The R Foundation for Statistical Computing, Vienna, Austria). A Benjamini-Hochberg adjust *p*-value of lower than 0.05 was considered statistically significant. The Gene Ontology (GO) annotations was performed by PANTHER analysis tool and Search Tool for the Retrieval of Interacting Genes/Proteins (STRING). Classification of protease and protease inhibitors was performed using PANTHER analysis tool and then manually confirmed by uniprot database.

### Western blot analysis

Fifty micrograms of CVF proteins were loaded onto a 4–12% Bis-Tris Mini Gel (Invitrogen, Carlsbad, CA) and transferred to a polyvinylidene difluoride (PVDF) membrane (Amersham Biosciences) by Mini Trans-Blot Cell system (Bio-Rad). The membrane was incubated in blocking solution (5% skim milk in TBS-T) for 30 min and then incubated with following primary antibodies: anti-SERPINB7 (Abcam, #ab127752) and anti-SOD1 (Abcam, #ab13498). After washing with TBS-T, membranes were incubated with appropriate secondary HRP-conjugated antibodies. Protein bands were visualized with a reagent from the Super Signal West Femto Maximum Sensitivity Substrate kit (Thermo Fisher Scientific) using chemiluminescence (Bio-Rad). Coomassie brilliant blue R-250 solution (Biosolution, #BC006b) was used to stain total proteins in the SDS-PAGE gel. The intensities of protein bands were analyzed using the ImageJ program (National Institutes of Health, Bethesda, Maryland).

## Results and discussion

### Study design

A total of 62 pregnant women participated in the study and CVF samples were collected at 14–20 weeks of gestation. From this cohort, CVF samples from 20 women who delivered at term (control group) and 5 women who delivered at spontaneous preterm (PTB group) were analyzed using a proteomic approach (see Experimental Section for details and Fig.1). Demographic characteristics of subjects are shown in Table 1.

**Table 1:** Demographic characteristics of subjects

	Delivery at term (*n* = 20)	Delivery at preterm (*n* = 5)	*P*-value
Maternal age (years)	33.25 ± 2.81	30.8 ± 2.59	0.115
BMI before pregnancy (kg/m^2^)	22.81 ± 3.65	22.8 ± 5.38	0.454
Gestational age at sampling (weeks)	17.19 ± 1.77	19.14 ± 1.66	0.071
Gestational age at delivery (weeks)	39.26 ± 1.02	31.57 ± 5.43	0.000

Values are expressed as means ± standard deviation

***Abbreviation*:** *n* number of subjects, *BMI* body mass index

We performed two independent iTRAQ experiments to compare the CVF proteome of individual PTB and individual control (term birth) subjects (Fig. 1b). The pooled CVF sample obtained from 20 individual term birth subjects was used as a spike-in reference standard for both Experiment 1 and 2.

### Proteomic profiling of CVF from pregnant women

**Fig. 2:**
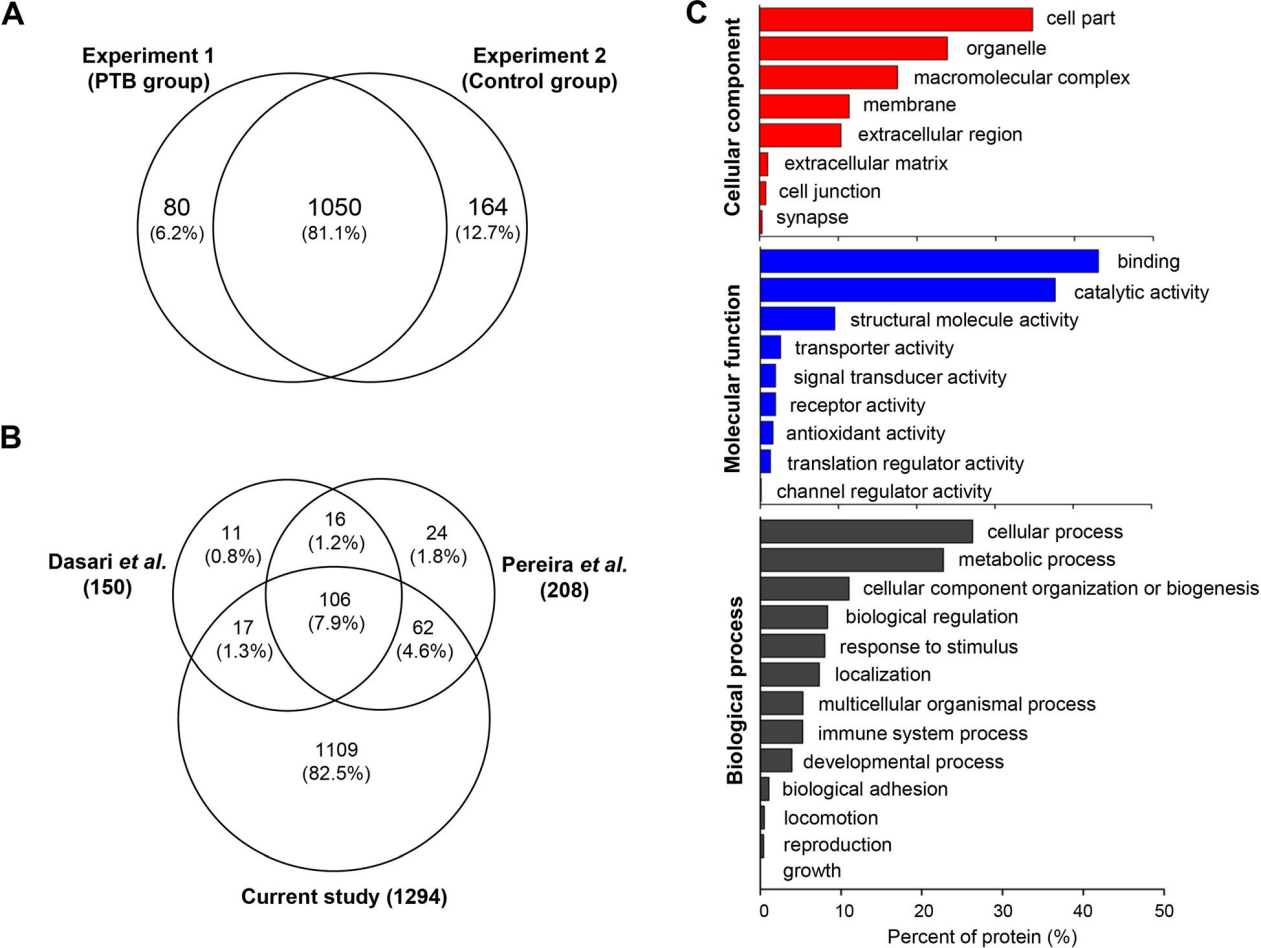
Proteomic profiling of CVF **a** Venn diagram displaying the number of CVF proteins identified in PTB (Experiment 1) and control group (Experiment 2). **b** Venn diagram illustrating the overlap of protein identifications between the present study and two previous CVF proteomic studies from pregnant women. **c** Functional classification of identified CVF proteins according to cellular component (red), molecular function (blue) and biological process (dark gray)

A total of 1294 proteins were identified with a peptide and proteins FDR of 0.01 when combining the results of both Experiment 1 and 2 (Fig. 2a and Table S1 in Supplementary Information). We compared our results to previously published proteomic analyses of human CVF. In total, 1875 unique CVF proteins have been identified in 12 previous studies so far (listed in Table S2 in Supplementary Information) [27, 33–43]. A majority of CVF proteomic profiling studies were performed in non-pregnant women [33, 35, 36, 39–43], while few studies reported CVF protein profiles of pregnant women [27, 34, 37, 38]. In this study, 605 proteins were not identified in previously published CVF proteomes of both pregnant and non-pregnant women, resulting in the total number of identified CVF proteins to 2480 (listed in Table S2 in Supplementary Information). We compared our proteomic data with two previously published CVF proteomic data from pregnant women (Fig. 2b and Fig. S1 in Supplementary Information) [27, 34]. These previous studies reported a relatively small number of CVF proteins identified in comparison to our proteomic data (150 and 208 proteins, respectively). A total of 106 proteins were shared by three experiments, which corresponds to approximately 70.6 and 50.9% of the total proteins identified in the study of Dasari et al. and Pereira et al., respectively. The overlap of protein identification between our data and three major CVF proteomic data from non-pregnant women [35, 39, 42] is also shown in Fig. S2 (Supplementary Information). Muytjens et al. reported a total of 1087 CVF proteins, of which 461 proteins (42.4% of total) were shared with our proteomic data.

The identified CVF proteins were categorized according to cellular component, molecular function and biological process using the PANTHER Classification System (Fig. 2c). The CVF proteins were mainly localized in the cell part (34.6%), organelle (23.8%), macromolecular complex (17.5%), membrane (11.3%) and extracellular region (10.3%). About 23% of identified CVF proteins were categorized in membrane and extracellular proteins. A total of 128 extracellular proteins were identified in this study, which is more than the number of extracellular proteins identified in previous studies [35, 39, 42]. For category of molecular function, a majority of proteins were associated with binding (43.2%), catalytic activity (37.7%) and structural molecule activity (9.5%). The biological process represented by CVF proteome included cellular (26.2%) and metabolic process (22.6%), cellular component organization (10.9%) and biological regulation (8.3%).

### Quantitative proteomic analysis of CVF from pregnant women with preterm or term birth

**Fig. 3:**
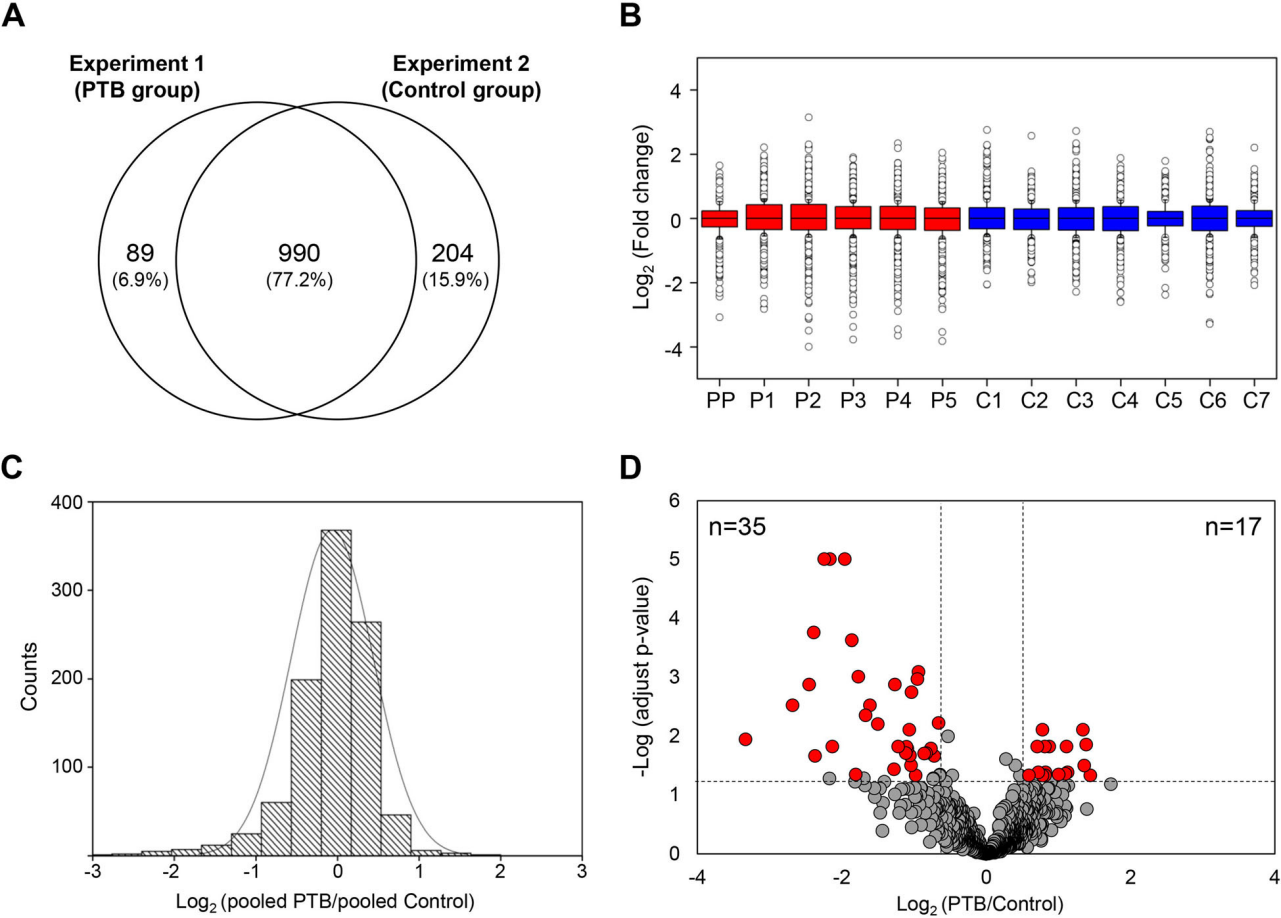
Quantitative proteomic comparison of CVF between PTB and control group. **a** Venn diagram illustrating the overlap of protein quantified in PTB (Experiment 1) and control (Experiment 2) group. **b** The box plot of normalized log_2_ iTRAQ ratios for PTB group versus pooled control (115/114, 116/114, 117/114, 118/114, 119/114 and 121/114) and control group versus pooled control (113/121, 114/121, 115/121, 116/121, 117/121, 118/121 and 119/121). A line across the box represents the median and outliers correspond to log_2_ values greater than 0.58 or less than − 0.58. PP, pooled PTB; P, PTB; C, control **c** The histogram distribution of normalized log_2_ iTRAQ ratio between pooled PTB and pooled control **d** A volcano plot of significantly changed proteins between PTB and control group. A plot is constructed from log_2_ fold-change (x-axis) and – log Benjamini-Hochberg adjust *p*-value (y-axis) for iTRAQ data of PTB and control group. The threshold for determining significantly differential expression is indicated by dashed lines (adjust *p*-value < 0.05, fold-change > 1.5). Dots selected in red indicate significantly up- and down-regulated proteins

Of 1283 quantified CVF proteins, 990 were commonly quantified in PTB (Experiment 1) and control (Experiment 2) group (Fig. 3a and Table S3 in Supplementary Information). The 6 iTRAQ ratios of PTB to pooled control (115/114, 116/114, 117/114, 118/114, 119/114 and 121/114) were calculated in PTB group. In control group, the 7 iTRAQ ratios of control to pooled control (113/121, 114/121, 115/121, 116/121. 117/121, 118/121 and 119/121) were calculated. The overall quality of the quantitative data was assessed with box plots and histogram of iTRAQ ratio distribution. Box plots showed a similar distribution of the normalized log_2_ iTRAQ ratios between PTB and control group (Fig. 3b). Figure 3c showed the histogram of the normalized log_2_ iTRAQ ratios for pooled PTB versus pooled control (115:114 ratio in Experiment 1), which followed a normal distribution.

To compare the CVF proteome of PTB versus control group, significant difference in protein abundance was determined based on the fold-change cut off of 1.5. The statistical significant threshold was set at a Benjamini-Hochberg adjust *p*-value < 0.05. As a result, 52 proteins were significantly changed between PTB and control, of which 17 proteins were up-regulated and 35 proteins were down-regulated in PTB compared to control (Fig. 3d). The list of significantly changed proteins are shown in Table 2 and Table S4 in Supplementary Information. Among these, serotransferrin (TF) was previously identified as a proteomic biomarker in CVF and serum [27, 44, 45]. In addition, angiotensinogen (AGT), ceruloplasmin (CP) and alpha-1B-glycoprotein (A1BG) have also been proposed as serum biomarkers for PTB [44, 45].

**Table 2:** List of differentially expressed proteins between PTB and control group

	Gene symbol
Up-regulated proteins in PTB	ZNF185, TRIM33, IGHV4–39, TOLLIP, TF*, S100A10, RPLP2, IMPA1, CP, METAP2, BST1, HSPA6, LRRFIP1, RAC1, A1BG, IGHV1–8, AGT
Down-regulated proteins in PTB	VAMP8, SDCBP2, FCAR, CREG1, ACP1, GLUL, CLCA4, SERPINB7, S100P, DMBT1, DPP4, KRT14, SERPINB2, EPS8L2, DSTN, GNB1, TP53I3, ARSB, KRT17, TGM3, DCD, RPTN, APEX1, GDF15, CCT2, SYNCRIP, FLG2, CPM, KRT2, KRT9, SPRR2D, KRT10, FLG, KRT1, LCE3D

**Notes:** Up/down-regulated proteins are determined with 1.5-fold changes and adjust *p*-value < 0.05. * Proteins that identified potential CVF biomarker for PTB in previous studies (Table S4 in Supplementary Information)

***Abbreviation*:** *PTB* preterm birth

**Fig. 4:**
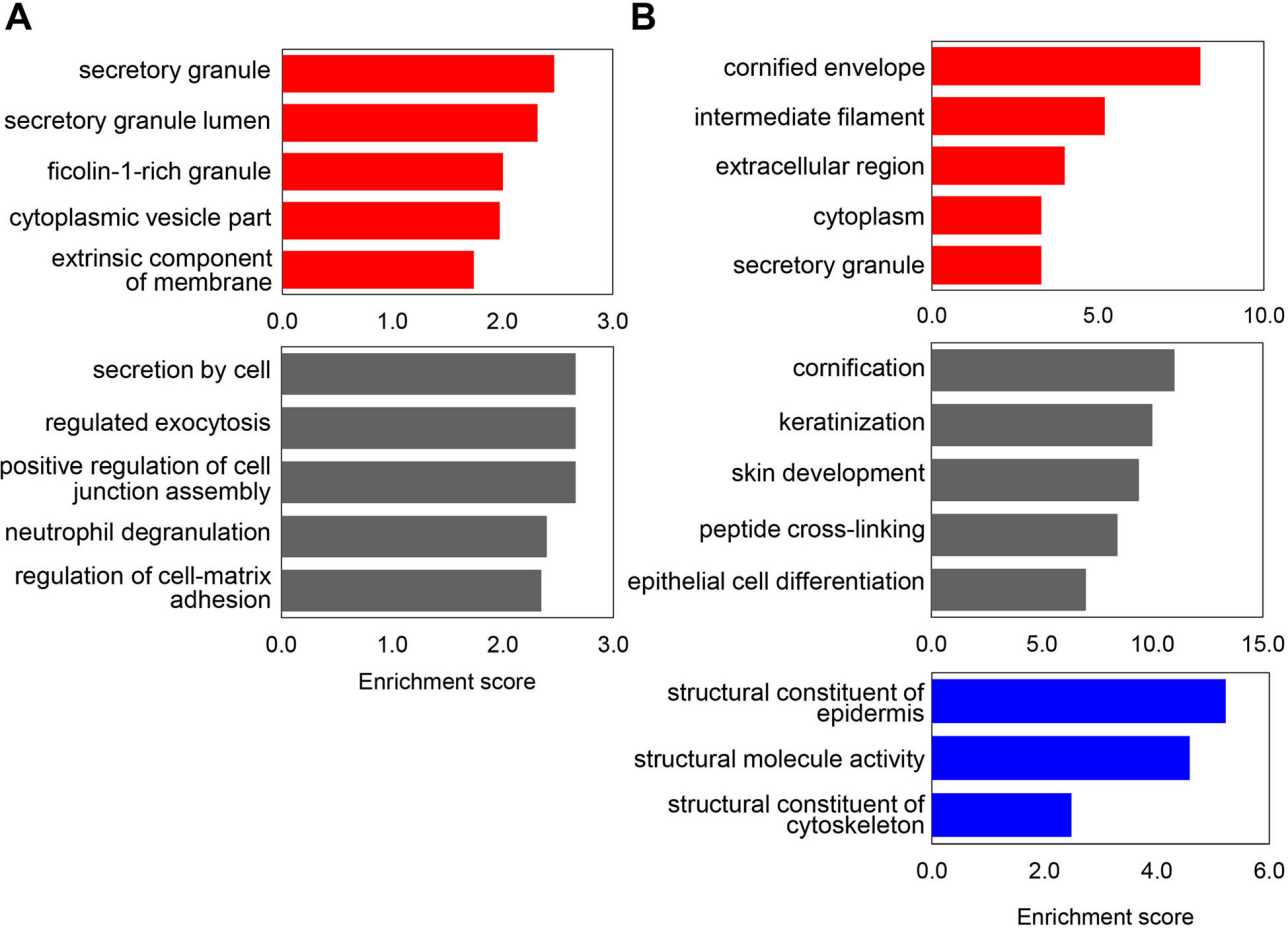
The top 5 GO terms enriched by differentially expressed proteins between PTB and control group. The up-regulated **a** and down-regulated **b** proteins were classified according to their cellular component (red), biological process (dark gray) and molecular function (blue). The enrichment score of GO terms was calculated by –log *p*-value

We further analyzed the GO enrichment to functionally characterize the significantly changed proteins between PTB and control. The proteins were classified into the cellular component, molecular function and biological process. The top 5 GO terms enriched by differentially expressed proteins are shown in Fig. 4. For the category of cellular component, up-regulated proteins were mainly located in secretory granule and lumen, and ficolin-1-rich granule and down-regulated proteins in cornified envelop, intermediate filament and extracellular region. The GO terms of molecular function including structural constituent of epidermis, structural molecule activity and structural constituent of cytoskeleton were enriched in down-regulated proteins. However, there was no significant enrichment of the molecular functions for up-regulated proteins. For biological process, up-regulated proteins were mainly enriched in the process of secretion by cell, regulated exocytosis and positive regulation of cell junction assembly, while proteins related to cornification, skin development, and epithelial cell differentiation were down-regulated in PTB.

### Inflammation-associated proteins

**Fig. 5:**
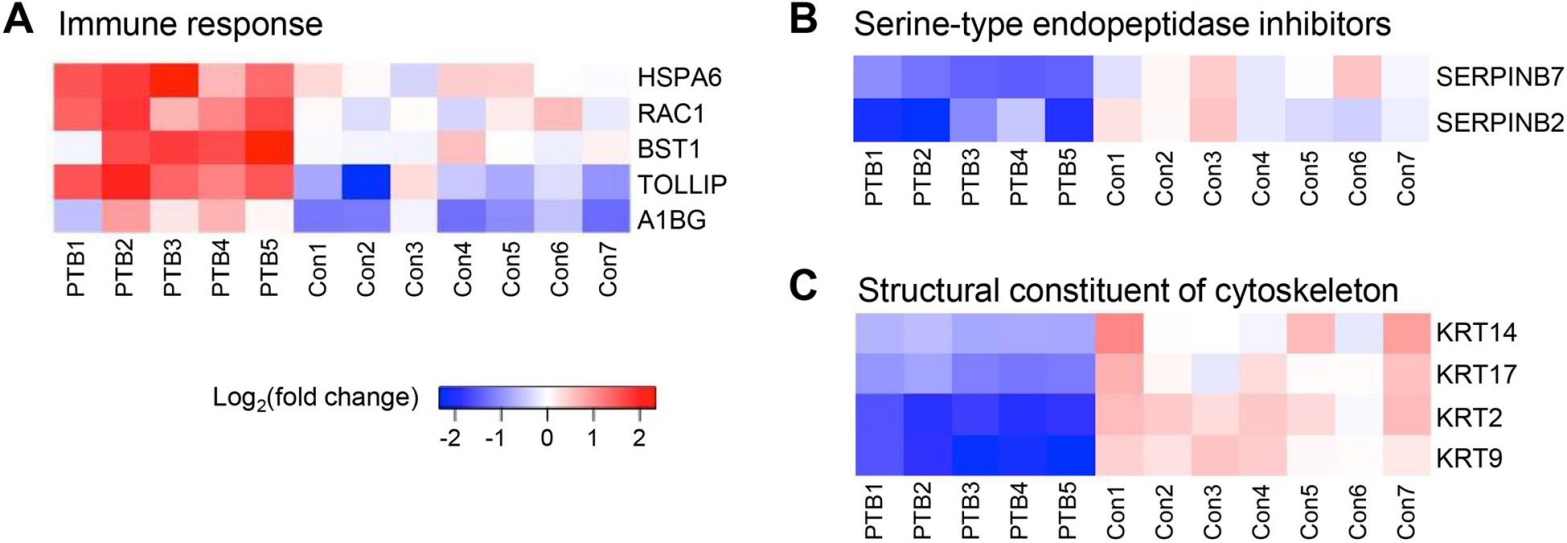
Heat map analysis of differentially expressed proteins between PTB and control group. Relative expression levels of proteins involved in immune response **a**, serine-type endopeptidase inhibitors **b** and structural constituent of cytoskeleton **c** were shown. The color scale illustrates the relative expression level of each protein. Red indicates up-regulation and blue indicates down-regulation

Inflammation is a key regulator of parturition process that triggers uterine contractility, cervical ripening and rupture of fetal membrane [46]. PTB is also considered to be closely related to inflammation, even though it is not infection-related PTB [47, 48]. Our proteomic results showed that up-regulated proteins are mainly localized in secretory vesicle (A1BG, BST1, HSPA6, RAC1, TF and TOLLIP) and ficolin-1-rich granule (A1BG, HSPA6 and RAC1), which are functionally enriched in neutrophil degranulation (A1BG, BST1, HSPA6, RAC1 and TOLLIP) (Fig.5). Neutrophils are effector cells of innate immune response, which release pro-inflammatory molecules by degranulation [49, 50]. The number of neutrophils increases during process of normal term parturition, resulting in increased inflammation [51, 52]. In addition, several previous studies reported that neutrophils are associated with inflammation-induced preterm birth in mice [51, 53]. Taken together, it is possible that neutrophilic inflammation contributes to preterm birth.

### Serine-type Endopeptidase inhibitors

Proteolytic activity is emphasized as one of the important functions of CVF proteins [54]. Muytjens et al. recently have identified a significant number of proteases and protease inhibitors in CVF proteome [42]. They found 85 proteases and 61 protease inhibitors (approximately 7.8 and 5.6% of identified CVF proteins, respectively) in CVF, which included 38 serine proteases (approximately 45% of identified protease). Our profiling data of CVF proteome showed 124 proteases and 53 protease inhibitors identified in CVF (approximately 9.5 and 4% of identified CVF proteins, respectively) (Table S5 and S6 in Supplementary Information). Of identified protease in CVF, serine proteases were most abundant group (approximately 62.9% of identified protease), followed by metalloprotease and cysteine proteases.

Interestingly, we observed that two serine-type endopeptidase inhibitors (SERPINB7 and SERPINB2) were significantly enriched in PTB-down-regulated proteins (Fig. 5), which indicated that the proteolytic activity of protease was aberrantly increased in CVF derived from PTB. The increased activity of protease may induce the deconstruction of extracellular matrix (ECM) at maternal-fetal interfaces or fetal membranes.

### Cytoskeletal proteins

Cytoskeletal proteins (e.g. fFN) are rarely detected in CVF due to the tight interactions of cells at maternal-fetal interfaces after 24 weeks of gestation [8]. However, cytoskeleton reorganization of uterine cervical epithelial cells occurs during early stages of pregnancy (before 20 weeks of gestation) [55, 56], therefore it is not an unexpected result that cytoskeletal proteins were observed in this study. Indeed, FN was detected in CVF from both PTB and control group, but there was no difference in abundance between both PTB and control group (PTB/control ratio, 1.309; adjust *p*-value, 0.408) (Table S3 in Supplementary Information).

We found that proteins involved in structural constituent of cytoskeleton (KRT2, KRT9, KRT14 and KRT17) were down-regulated in PTB compared to control. GO terms of biological process including skin development and epidermis differentiation were also enriched in PTB-down-regulated proteins (Fig. 4). Keratins are generally excluded from LC-MS/MS data because it is considered as common contaminants. However, since keratins are most abundant structural proteins in epithelial cells (e.g. cervical and vaginal mucosal epithelia) [57–59], these proteins also could be potential CVF biomarkers for the prediction of PTB in asymptomatic women. In previous studies, keratin, type 1 cytoskeletal 19 (KRT19) was identified as a biomarker for PTB in amniotic fluid and placental tissue [60, 61]. The down-regulation of cytoskeletal proteins in PTB compared to control possibly contributed to the incomplete formation of maternal and fetal membranes.

### Verification of the significant changed protein between PTB and control group by Western blot analysis

**Fig. 6:**
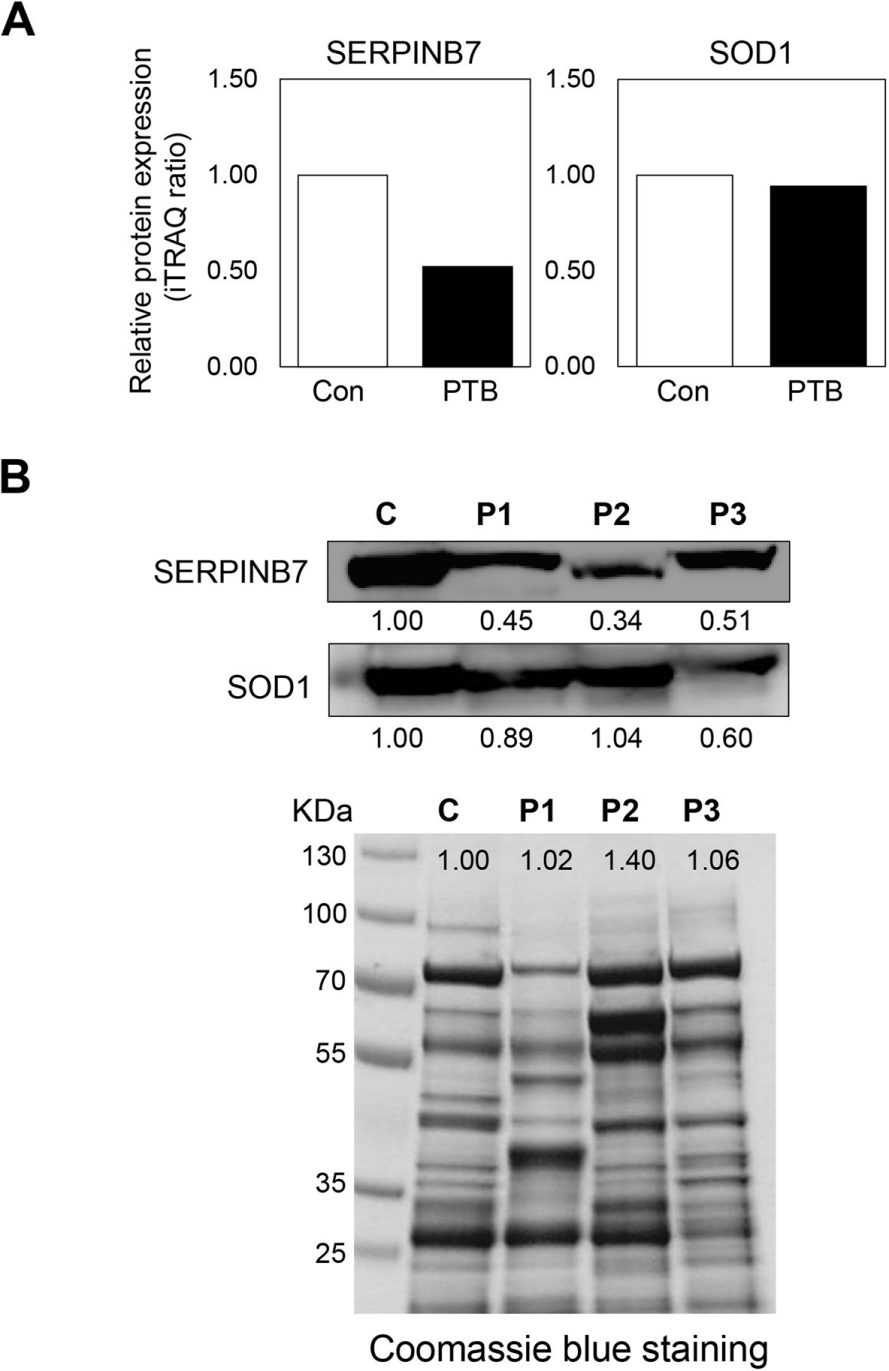
Validation of SERPINB7 by Western blot analysis. **a** Relative protein expression level of SERPINB7 and SOD1 from iTRAQ data. Protein expression levels were normalized to control. **b** Validation of SERPINB7 (down-regulated protein in PTB) and SOD1 (unchanged protein in this study) in both PTB and control samples by Western blot. C, pooled control from 4 control CVF; P, preterm birth CVF

We performed Western blot analysis in PTB and control group to verify iTRAQ-based proteomic data. Out of up/down-regulated proteins in PTB, we quantified SERPINB7 (a serine-type protease inhibitor) that was a down-regulated protein in PTB compared to control group (Fig. 6a). In addition, SOD1 (an unchanged protein between two group in this study) was also quantified in PTB and control group. Resultingly, the expression levels of SERPINB7 in PTB were down-regulated compared control and also well match with proteomic assessments (Fig. 6b). There was no noticeable difference in the level of protein expression between Control and PTB.

## Conclusion

We have explored the proteomic profiles of CVF from pregnant women who ultimately delivered at preterm and term. We identified 1294 CVF proteins that include a number of newly identified proteins, resulting in expanded the CVF proteome. Our results also unveiled that proteins involved in immune response, structural constituent of cytoskeleton and negative regulation of serine-type protease were significantly changed in PTB compared to control group. Finally, we verified the down-regulation of SERPINB7 by Western blot analysis. This study was conducted in a relatively small number of subjects due to the difficulty in obtaining CVF related to PTB, further research is necessary to validate these potential biomarkers in a large cohort study.

## Supplementary Information

**Additional file 1: Table S1**: List of all identified proteins in CVF. **Table S2**. List of all identified CVF proteins in previous proteomics studies and this study. **Table S3**. List of all quantified proteins in CVF. **Table S4**. List of significantly up−/down-regulated proteins in PTB compared to control group. **Table S5**. List of proteases identified in CVF. **Table S6**. List of protease inhibitors identified in CVF. **Table S7**. List of raw data files from LC-MS/MS experiments.

**Additional file 2: Figure S1.** Comparison of the present study and two previous CVF proteomic studies from pregnant women. **Figure S2.** Venn diagram illustrating the overlap of protein identifications between the present study and three previous CVF proteomic studies from non-pregnant women.

## Data Availability

Excel file containing the analyzed data are provided in Supplementary Information. The datasets generated via nLC-ESI-MS/MS analyses in this study are available in PRIDE, accession number: PXD021401. https://www.ebi.ac.uk/pride/archive/login.
