# Supplementary material for: Quantitative proteomic profiling of Cervicovaginal fluid from pregnant women with term and preterm birth
Source: Proteome Sci. 2021 Feb 15;19:3. doi: 10.1186/s12953-021-00171-1 (PMC7885372; doi:10.1186/s12953-021-00171-1)
Supplement: Supplementary file 2 — Additional file 2: Figure S1. Comparison of the present study and two previous CVF proteomic studies from pregnant women. Figure S2. Venn diagram illustrating the overlap of protein identifications between the present study and three previous CVF proteomic studies from non-pregnant women. [file 12953_2021_171_MOESM2_ESM.docx]

**Supplementary Information**

Quantitative Proteomic Profiling of Human Cervicovaginal Fluid for Early Detection of Preterm Birth

Young Eun Kim,^1,§^ Kwonseong Kim,^1, 2,§^ Han Bin Oh, ^2^ Myoung Seng Kwon,^3^ Sung Ki Lee, ^3,*^ Dukjin Kang^1,*^

^1^Center for Bioanalysis, Division of Chemical and Medical Metrology, Korea Research Institute of Standards and Science, Daejeon, 34113, Korea

^2^Department of Chemistry, Sogang University, Seoul, 04107, Korea

^3^Department of Obstetrics and Gynecology, Konyang University Hospital, Daejeon, 35365, Korea

^*^Corresponding author: Dukjin Kang

Center for Bioanalysis

Korea Research Institute of Standards and Science

267 Gajeong-Ro, Yuseong-Gu,

Daejeon, 34113, Korea

phone: (82) 42 868 5700

fax: (82) 42 868 5801

e-mail: [djkang@kriss.re.kr](mailto:djkang@kriss.re.kr)

^*^Co-corresponding author: Sung Ki Lee

Department of Obstetrics and Gynecology

Konyang University Hospital

158 Gasuwondong-Ro, Seo-Gu,

Daejeon, 3535, Korea

phone: (82) 42 600 9204

fax: (82) 42 600 9760

e-mail: [sklee@kyuh.ac.kr](mailto:sklee@kyuh.ac.kr)

^§^Both authors contributed equally to this study

**Supplementary Figures**

**Figure S1.** Comparison of the present study and two previous CVF proteomic studies from pregnant women.

**Figure S2.** Venn diagram illustrating the overlap of protein identifications between the present study and three previous CVF proteomic studies from non-pregnant women.

**Supplementary Tables**

All tables listed here are shown in a supporting Excel file.

**Table S1.** List of all identified proteins in CVF

**Table S2.** List of all identified CVF proteins in previous proteomics studies and this study.

**Table S3.** List of all quantified proteins in CVF

**Table S4.** List of significantly up-/down-regulated proteins in PTB compared to control group

**Table S5.** List of proteases identified in CVF

**Table S6.** List of protease inhibitors identified in CVF

**Table S7.** List of raw data files from LC-MS/MS experiments

**Supplementary Figures**

**
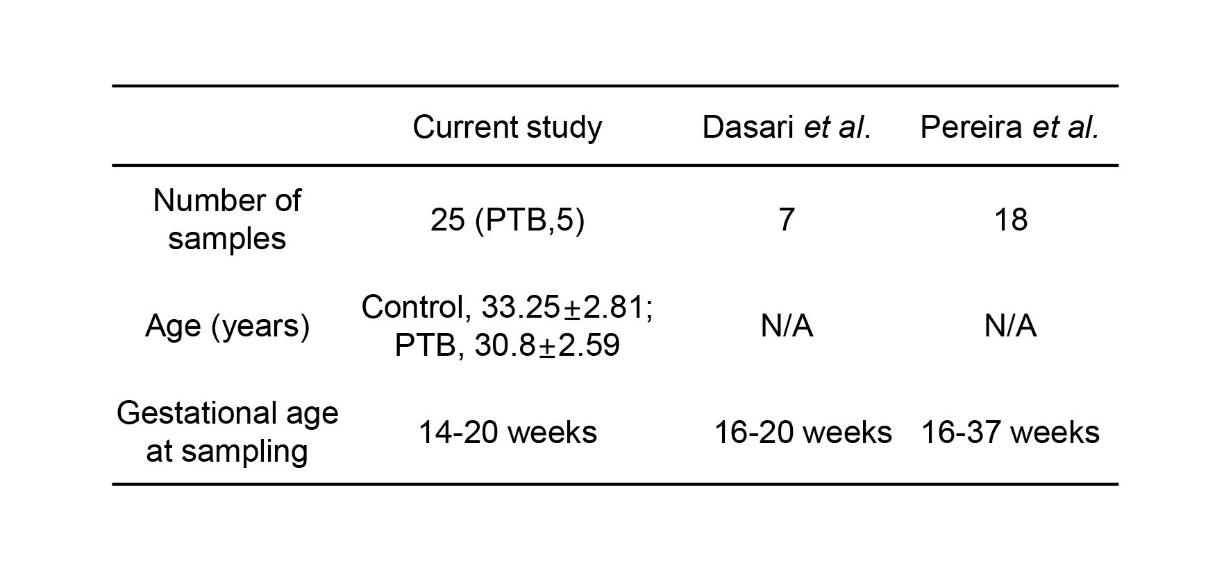
**

**Figure S1.** Comparison of the present study and two previous CVF proteomic studies from pregnant women

**
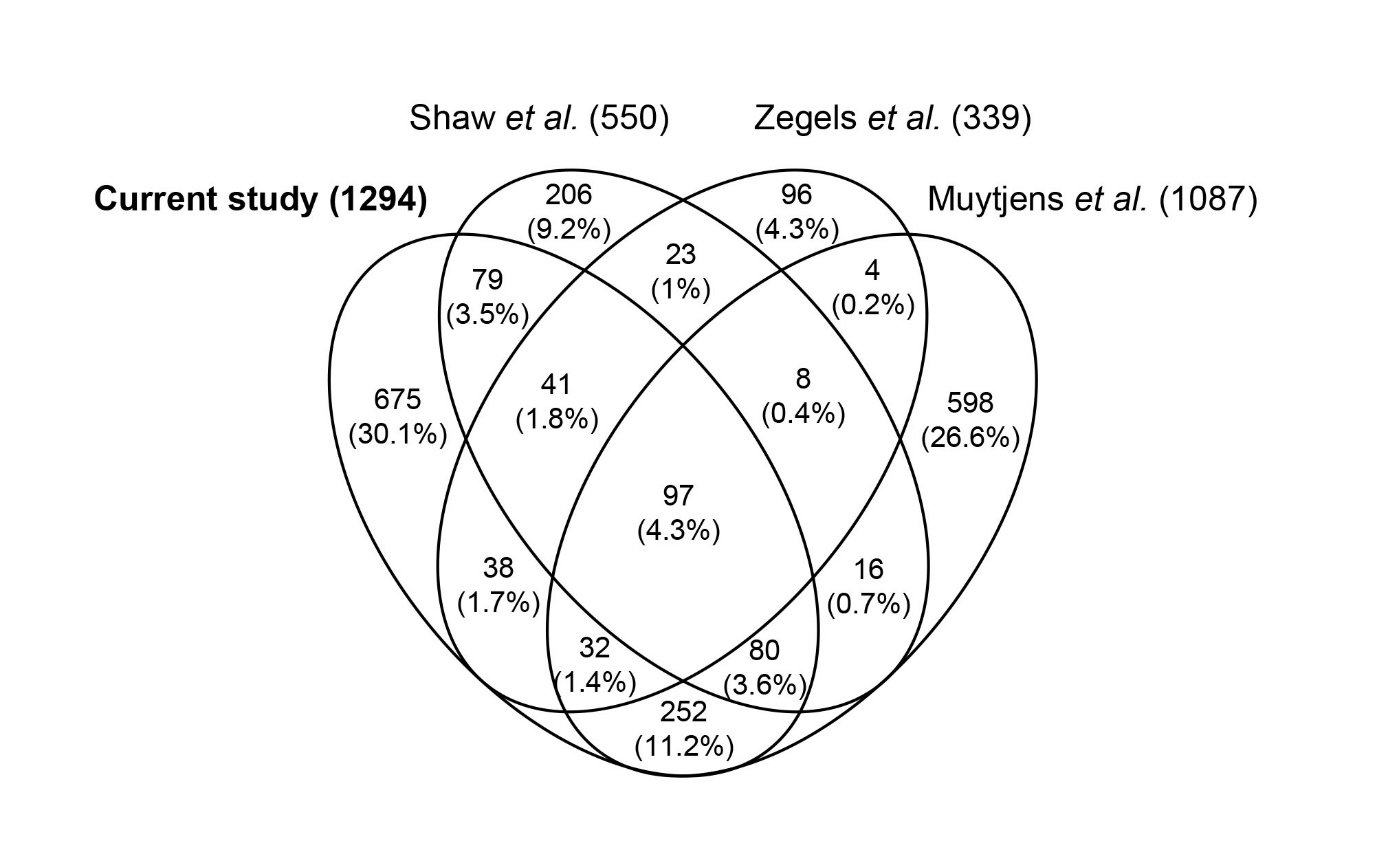
**

**Figure S2.** Venn diagram illustrating the overlap of protein identifications between the present study and three previous CVF proteomic studies from non-pregnant women.
